# Mouth Conditions as Related to the Physician’s Work

**Published:** 1916-10

**Authors:** C. M. M’Cauley

**Affiliations:** Dallas, Texas


### Mouth Conditions as Related to the Physician's Work.

BY C. M. M'CAULEY, B. S., D. D. S., DALLAS, TEXAS.

### MOUTH TROUBLES OF THE CHILD.

The average normal child at birth weighs seven and a half pounds and is twenty inches in length. If development is allowed to progress unhampered the child at the end of the first year has gained in weight twelve and a half pounds and is twenty-nine inches long. Let us see why many children do not develop in this way; at least, let us observe the mouth conditions which assist in retarding development. Assuming that the child has the benefit of food, rest and good air to breathe, one of the first obstacles in the way of development is the result of colds. Frequent colds cause the growth of adenoids and the presence of tonsilitis. The normal child uses the nose as a breathing organ, the mouth remaining closed during the act of breathing. When the nasal passages are partly closed, or wholly closed by adenoids, the child is compelled to open the mouth to breathe, and this is the beginning of mouth troubles.

### HOW MOUTH BREATHING AFFECTS THE DENTAL ARCHES.

As stated above, the normal child breathes through the nose, the mouth being closed. When the mouth is closed there is an equilibrium established by the weight of the cheeks pressing inward and the tongue pressing outward, the result being the eruption of the teeth around the border of the tongue and just inside the cheeks. These two forces are the most prominent influences in lining the teeth properly in the arches. If perfect arches are to be formed, these forces must remain undisturbed. If disturbance of these forces takes place the arches will not be perfect, for the following reasons:

When the mouth is closed the tongue occupies the proper position to produce normal eruption of the teeth. When it is open the tongue drops into the back of the mouth and the pressure upon the ridges or alveoli from the inside is diminished or entirely lost. At the same time the muscles of the cheeks, instead of exerting a normal pressure from the outside, are in a distended position and the pressure is very much greater. With the inside force gone and the outside force increased, it is not difficult to see that the result will be the pressing together of the alveolar ridges of both the upper and lower jaws, resulting in a narrowed arch above and below, and raising the vault of the mouth. When the vault is raised, the floor of the nose is also raised, and thus nasal breathing becomes more and more obstructed. In these cases of high vault it is possible that the raising of the vault will exert pressure upon the floor of the brain cavity, raising it slightly, thereby lessening the size of the brain cavity. The mental effect may be marked.

Associated with the above condition of nose, throat and mouth will be found a flattened chest and rounding shoulders. The child is poorly fortified for defense against the many ills to which it is subjected at this age. Its vitality is lowered and its resistance very much impaired, rendering it more susceptible to the encroachment of disease.

Please do not understand that these conditions come about suddenly. They begin early in life, during the first year many times, and the process of damage may be rapid or it may require several years to note such results as the narrowed arch, flattened chest and rounded shoulders.

At about the age of four years the baby teeth begin to separate in the normal mouth. If this fails to occur, we should take warning that something is wrong. Development of the child is not what it should be. We should be very watchful of the baby teeth, because they are a perfect index as to what the permanent teeth will be; that is, their position in the arches. If the growth and development of the jaws is not such as to cause the spaces to occur between the baby teeth at about four years of age or very soon thereafter, we may expect the permanent teeth to be irregular and badly out of line. No one will question the wisdom on the part of the physician who has supervision over the health of the little one at this age who observes closely the spacing of the teeth and refers to the mouth as a very important index for the normal or abnormal development of the child. Before the seventh year is the best time for the orthodontist to see these cases for the reason that a slight enlargement of the arch at this age is very much easier than in later years, after the permanent teeth have erupted in wrong positions. It means simply the prevention of irregularity in the permanent set of teeth, as well as the restoration of breathing space in the nasal cavities by lowering the vault to its normal height, and making it possible for the child to masticate its food.

Many parents and some physicians regard the first teeth as of little value because they are to be lost in a few years and replaced by a permanent set, so they are allowed to decay and be lost without any knowledge as to the damage done. Let me say in this connection that if I were comparing the value of healthy, normal baby teeth, and the importance of preventing decay and preserving them with the value of permanent teeth, I should place the care of the baby teeth ahead of the permanent ones, because healthy, normal conditions up to the seventh year is the very best assurance of good permanent teeth, while decay and loss of baby teeth will surely lead to abnormal conditions in the next set. No baby tooth should ever be extracted until it naturally becomes loose enough to remove with a string. Any exception to this rule is the result of an abnormal condition which could have and should have been prevented.

The observing physician should discover such symptoms as mouth-breathing in infants and children, failure of the baby teeth to separate from four to six or seven years of age, and by early treatment prevent a multitude of ill results which follow. Mouth-breathing should be stopped by removing the cause and placing adhesives over the mouth. Failure of the teeth to space properly should have the attention of the orthodontist.

[The next paper will deal with the importance of proper mastication and how it can be produced.]

---

An old darkey appeared in the doctor's office one morning, plainly very low in his mind. The doctor, recognizing his old patient, greeted him in his most inspiriting manner.

"Well, Elijah, how is the rheumatism these days?"

"Porly, porly, sah!" replied Elijah dejectedly. "Belieb me, Marse Doctor, I'se jest a movin' picture ob pain!"—*Woman's Companion.*

---

### Do You Know That?

The Constitution of the United States doesn't mention health?

Procrastination in sanitary reform is the thief of health?

A book on "Exercise and Health" may be had free for the asking from the United States Public Health Service?

Not everybody can achieve greatness, but everybody can be clean?

If you sow a hygienic habit you reap health—reap health and you attain longevity?

Railway cars would be sanitary if it weren't for the people in them?

America's typhoid fever bill is more than $270,000,000 a year?

The full dinner pail is the enemy of tuberculosis?

